# *Cronobacter sakazakii*, *Cronobacter malonaticus*, and *Cronobacter dublinensis* Genotyping Based on CRISPR Locus Diversity

**DOI:** 10.3389/fmicb.2019.01989

**Published:** 2019-08-28

**Authors:** Haiyan Zeng, Chengsi Li, Wenjing He, Jumei Zhang, Moutong Chen, Tao Lei, Haoming Wu, Na Ling, Shuzhen Cai, Juan Wang, Yu Ding, Qingping Wu

**Affiliations:** ^1^State Key Laboratory of Applied Microbiology Southern China, Guangdong Provincial Key Laboratory of Microbiology Culture Collection and Application, Guangdong Open Laboratory of Applied Microbiology, Guangdong Institute of Microbiology, Guangdong Academy of Sciences, Guangzhou, China; ^2^College of Food Science, South China Agricultural University, Guangzhou, China; ^3^Department of Food Science and Technology, Jinan University, Guangzhou, China

**Keywords:** *C. sakazakii*, *C. malonaticus*, *C. dublinensis*, CRISPR typing, multi-locus sequence typing, whole genome sequence typing

## Abstract

*Cronobacter* strains harboring CRISPR-Cas systems are important foodborne pathogens that cause serious neonatal infections. CRISPR typing is a new molecular subtyping method to track the sources of pathogenic bacterial outbreaks and shows a promise in typing *Cronobacter*, however, this molecular typing procedure using routine PCR method has not been established. Therefore, the purpose of this study was to establish such methodology, 257 isolates of *Cronobacter sakazakii*, *C. malonaticus*, and *C. dublinensis* were used to verify the feasibility of the method. Results showed that 161 *C. sakazakii* strains could be divided into 129 CRISPR types (CTs), among which CT15 (*n* = 7) was the most prevalent CT followed by CT6 (*n* = 4). Further, 65 *C. malonaticus* strains were divided into 42 CTs and CT23 (*n* = 8) was the most prevalent followed by CT2, CT3, and CT13 (*n* = 4). Finally, 31 *C. dublinensis* strains belonged to 31 CTs. There was also a relationship among CT, sequence type (ST), food types, and serotype. Compared to multi-locus sequence typing (MLST), this new molecular method has greater power to distinguish similar strains and had better accordance with whole genome sequence typing (WGST). More importantly, some lineages were found to harbor conserved ancestral spacers ahead of their divergent specific spacer sequences; this can be exploited to infer the divergent evolution of *Cronobacter* and provide phylogenetic information reflecting common origins. Compared to WGST, CRISPR typing method is simpler and more affordable, it could be used to identify sources of *Cronobacter* food-borne outbreaks, from clinical cases to food sources and the production sites.

## Introduction

The *Cronobacter* (formerly *Enterobacter sakazakii*) genus including *C. sakazakii, C. malonaticus, C. dublinensis, C. turicensis, C. universalis, C. muytjensii*, and *C. condimenti* comprises opportunistic foodborne pathogens that can cause rare but life-threatening diseases in neonates and immune-compromised infants, including meningitis, necrotizing enterocolitis, and septicemia (Iversen et al., 2008; Kucerova et al., 2011; Joseph et al., 2012a; Zeng et al., 2018a). Latest study reported an acute gastroenteritis outbreak caused by *C. sakazakii* in a senior high school of China (Yong et al., 2018). Moreover, this genus has been isolated from the environment, food, and clinical sources (Ueda, 2017; Yong et al., 2018; Zeng et al., 2018a; Li et al., 2019). Further, *C. sakazakii*, *C. malonaticus*, and *C. dublinensis* are three prevalent species and some reports have indicated that the principal sources of these organisms might be soil, water, and vegetables (Ueda, 2017; Zeng et al., 2018b). However, the epidemiology and reservoirs of *Cronobacter* spp. is still unsure (Holy and Forsythe, 2014).

Some molecular subtyping methods have been developed to study the epidemiology of pathogenic bacteria, including pulsed field gel electrophoresis (PFGE) and multi-locus sequence typing (MLST), but both still have some disadvantages (Ogrodzki and Forsythe, 2017). PFGE is limited for a portion of *Cronobacter* strains that cannot be typed due to intrinsic DNase activity; moreover, it does not provide the phylogenetic relationship between strains. MLST has been established for *Cronobacter* genus based on seven housekeeping genes (Joseph et al., 2012b). A curated open access MLST database has been established for the genus with more than 2200 strains and associated metadata^1^. This database has enabled the recognition of certain *Cronobacter* clonal lineages within the genus as pathogenic variants, whereas others are primarily commensal organisms associated with the environment. The discrimination power of MLST is weaker than that of whole genome sequence typing (WGST), and this method lacks information about historical ancestors (Forsythe et al., 2014). WGST is a new method for subtyping bacteria, but its high costs still limit its application (Forsythe et al., 2014; Deng et al., 2015).

CRISPR-Cas system is an adaptive immune system for bacteria, providing bacteria with sequence-specific, acquired defense against phages and plasmids (Barrangou, 2013; Westra et al., 2014). The evolution of CRISPR-Cas has led to the discovery of a diverse set of CRISPR-Cas systems, which can be then classified into distinct classes, types, and subtypes, combined with the analysis of signature protein families and features of *cas* loci architectures that unambiguously partition most CRISPR–Cas loci (Makarova et al., 2015; Shmakov et al., 2017). The activity of a CRISPR locus occurs in three stages as follows: adaptation through the incorporation of new spacers into the existing repeat-spacer array; expression of the repeat-spacer array and the consequent processing of that array into CRISPR RNAs (crRNAs); interference during which invasive target sequences are recognized and destroyed by the crRNA-effector complex (Barrangou et al., 2007). As new spacers are added to one end of the CRISPR array, polarity exists; specifically, spacers at the leader distal end are more ancient and are often shared among bacterial common ancestors. The acquisition, loss, and duplication of spacers have made CRISPR arrays be the fastest evolving loci in bacteria (Paez-Espino et al., 2013; Shariat and Dudley, 2014).

The first application of CRISPR loci in bacterial genotyping was spacer-oligonucleotide typing (or “spoligotyping”) of *Mycobacterium tuberculosis* strains (Groenen et al., 1993; Streicher et al., 2007). Its principle is PCR amplification of the CRISPR array with labeled primers that recognize the directed repeat sequences, then hybridization of the PCR products to a membrane containing probes bearing spacer DNA sequences (Streicher et al., 2007). The “next-generation” microbead-spoligotyping approach was an assay termed CRISPOL (for “CRISPR polymorphism”) applied to *Salmonella* (Fabre et al., 2012). The first application of sequence-based CRISPR typing was group A *Streptococcus* (GAS) M1 serotype (Hoe et al., 1999). Considered the temporal organization of spacers, the sequencing of CRISPR arrays has been a extremely useful tool to genotype bacteria like *Yersinia* species, *E. coli*, and *Salmonella enterica* (Cui et al., 2008; Fricke et al., 2011; Yin et al., 2013; Li et al., 2014; Bugarel et al., 2018), and it has also been used to investigate bacterial diversity based on metagenomic data (Berg Miller et al., 2012; Sun et al., 2016). Recently, some useful tools to extract spacers and visualize the spacer content with color schemes were developed (Biswas et al., 2016; Couvin et al., 2018; Dion et al., 2018; Nethery and Barrangou, 2019).

In previous studies, six CRISPR arrays were detected in conserved regions of the *Cronobacter* genomes; among these, CRISPR1 and CRISPR2 neighbor the I-E type of the “complete” *cas* gene cluster, whereas CRISPR3 and CRISPR6 integrate with the I-F type of the “complete” *cas* gene cluster comprising subtype I-E and I-F CRISPR-Cas systems, respectively. Two CRISPR-Cas systems (Subtype I-E and I-F) were found only in *C. sakazakii*, *C. malonaticus*, and *C. dublinensis* isolates, specifically. Unlike subtype I-E, which was commonly detected among *Cronobacter* strains, subtype I-F was found to be significantly more prevalent in the plant-associated species *C. dublinensis* than in the human virulence-related species *C. sakazakii* and *C. malonaticus*. However, *C. condimenti* lacked intact CRISPR-Cas system (Zeng et al., 2017, 2018b). At the same time, significantly higher CRISPR activity was also observed in the plant-associated species *C*. *dublinensis* than in the virulence-related species *C. sakazakii* and *C*. *malonaticus* (Zeng et al., 2018b). Similar CRISPR array spacers have been rarely detected among species, indicating intensive changes through adaptive acquisition and loss. Thus, differentiated CRISPR activity appears to be the product of environmental selective pressure and might contribute to the bidirectional divergence and speciation of *Cronobacter* (Zeng et al., 2018b).

CRISPR arrays will be a promising typing method compared with MLST (Ogrodzki and Forsythe, 2016, 2017; Zeng et al., 2018b). However, the identification of CRISPR arrays in *Cronobacter* was based on whole genome sequences by next generation sequencing, which is associated with high cost. It is necessary to establish this new molecular subtyping method using routine PCR and define the nomenclature system. Among six types of CRISPR arrays, CRISPR1 and CRISPR2 were found in almost all *Cronobacter* strains, whereas CRISPR3 and CRISPR6 were also found to be preserved in many *Cronobacter* strains, and all of them have a high diversity among different isolates (Zeng et al., 2018b). However, CRISPR4 and CRISPR5 were not suitable for genotyping, as they were only found in a few *C. sakazakii* isolates and lacked spacer diversity (Zeng et al., 2017). The diversity of four CRISPR arrays in isolates of this genus could therefore provide a powerful tool to track the origin of genetically similar strains within an outbreak. In this study, we established a CRISPR-based subtyping method for *C. sakazakii*, *C. malonaticus*, and *C. dublinensis* using routine PCR and examined the relationship between CRISPR profiles and other genetic factors.

## Materials and Methods

### Bacterial Isolates

A total of 257 *Cronobacter* isolates used in this study were collected from four types of food (powdered infant milk, ready-to-eat food, vegetables, and edible mushroom) in China, including 161 *C. sakazakii*, 65 *C. malonaticus*, and 31 *C. dublinensis* strains. All strains belonged to the large-scale and systematic investigation on the prevalence of *Cronobacter* spp. in food in China, and detailed information about these strains, including O serotypes, STs, and antibiotic-resistance profiles, are provided in Supplementary Data Sheets 1–3 (Zeng et al., 2017, 2018b; Ling et al., 2018; Li et al., 2019).

### CRISPR PCR Amplification and Sequencing

**FIGURE 1:**
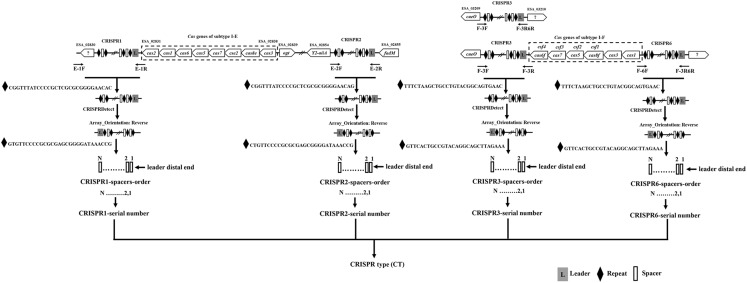
Outline of the new molecular typing method based on four CRISPR arrays of *Cronobacter*. The locations of PCR primers used to amplify CRISPR loci are shown. Compared to that in *C. sakazakii* and *C. malonaticus*, there was a 1-kb plus nucleotide sequence region including one hypothetical protein between the hypothetical protein used for the design of primer E-1F and CRISPR1 in *C. dublinensis* isolates. The orientation of CRISPR arrays and extraction of spacers were completed by CRISPRDetect. The specific CRISPR type was determined by a combination of sequenced incorporated spacers in CRISPR arrays.

The primers location for the amplification CRISPR1, CRISPR2, CRISPR3, and CRISPR6 are shown in Figure 1, and are in accordance with the genomic sequences encoding CRISPR-Cas systems in *Cronobacter* reported previously (Zeng et al., 2018b). The sequences of primers for the amplification and sequencing of CRISPR1, CRISPR2, CRISPR3, and CRISPR6 loci are listed in Table 1. PCR reaction was performed using a 50-μL volume, which contained 0.5 μL of PrimeSTAR^®^HS DNA Polymerase (2.5 U/μL; Takara, Dalian, Japan), 4 μL of 2.5 mM dNTPs, 0.5 μL of each 10 mM primer, 10 μL of 5 × PrimeSTAR Buffer, and 1 or 2 μL of bacterial DNA template for CRISPR1 and CRISPR2 or CRISPR3 and CRISPR6, respectively; the remaining volume consisted of sterile water. The PCR conditions were as follows: initial denaturation at 98°C for 1 min; 30 cycles of 98°C for 10 s, 58°C for CRISPR1 and 57°C for CRISPR2, CRISPR3, and CRISPR6 for 5 s, and 72°C for 4 min; a final extension at 72°C for 5 min. After identification by electrophoresis, the PCR products were subjected to DNA sequencing directly (Beijing Genomics Institute, Guangzhou, China). All PCR products were sequenced using amplification primers in both the forward and reverse directions to obtain a double-stranded sequence. PCR products larger than 2-kb sometimes required the design of additional primers based on Sanger sequencing results by amplification primers to obtain the complete sequences.

**TABLE 1:** Primers for CRISPR1, CRISPR2, CRISPR3, and CRISPR6 loci.

**Sequence amplified**	**Species**	**Primers**	**Primer sequences (5′-3′)**
CRISPR1	*C. sakazakii; C. malonaticus; C. dublinensis*	E-1F	CCTGACCTGGTAAACAGAGTAGCG
		E-1R	CGATTTCCAGACGTWCGGCGTTAA
CRISPR2	*C. sakazakii; C. malonaticus; C. dublinensis*	E-2F	CAGTTRAGATGGTGTACYCGCATA
		E-2R	ARAGGGCAGCCGRTCTTTAACAAG
CRISPR3	*C. sakazakii; C. malonaticus*	E-3F	GTTGAGCTTAAACCCTCCCCTTGC
		E-3R	GTCAGCGGYACCTTCAGCAGTT
	*C. dublinensis*	E-3F	GTTGAGCTTAAACCCTCCCCTTGC
		E-3R-dub	TCTCTCCAGCGGCCAGTAYTACAG
CRISPR6	*C. sakazakii*	E-6F-sak	GTCAACTTTTATARGGCCTTCGC
		E-3R6R^∗^	CAGGCATTCCGGTAATATTCGCTC
	*C. malonaticus*	E-6F-mal	GCAATTAGCACCTGACTGATGTACG
		E-3R6R	CAGGCATTCCGGTAATATTCGCTC
	*C. dublinensis*	E-6F-dub	GGTATGGKCTTTTGCCTTCG
		E-3R6R- dub^#^	TACCGCCCGTCCTTAAGVTATTG

*^∗^If CRISPR3 from *C. sakazakii* and *C. malonaticus* was not amplified by E-3F and E-3R, then E-3R was replaced with E-3R6R. #If CRISPR3 from *C. dublinensis* was not amplified by E-3F and E-3R-dub, then E-3R was replaced with E-3R6R-dub.*

### CRISPR Typing and Cluster Analysis

The orientation of CRISPR spacers were determined by CRISPRDetect and the spacers were extracted using CRISPRCasFinder (Biswas et al., 2016; Couvin et al., 2018). A similarity search of the identified spacer sequences (84% similarity) and the establishment of a unique spacer library were performed as described previously (Zeng et al., 2017). A comparison of these unique spacers to previously studied elements within the ACLAME database (Leplae et al., 2010) was performed to identify potential targets. Every unique spacer among different CRISPR arrays of one species was assigned a single number beginning with 1 from the leader distal end, and lists of CRISPR spacer sequences for *C. sakazakii*, *C. malonaticus*, and *C. dublinensis* are provided as Supplementary Data Sheets 4–6, respectively. Then, every CRISPR array with multiple spacers was assigned a number as a spacer code. CRISPR typing was performed by combining CRISPR1, CRISPR2, CRISPR3, and CRISPR6 into one allele and displayed this as an arrangement of CRISPR spacers. The CRISPR type (CT) of each isolate was defined using a specific number to reflect its unique allelic type. The discrimination index (D) was calculated based on the Simpson’s index of diversity with the equation as previously defined (Hunter and Gaston, 1988). To depict the clustering of subtypes determined by CRISPR diversity, the binary distribution (presence as “1” or absence as “0”) of every spacer in each CRISPR locus was profiled for each strain. The binary distribution patterns of all strains were then combined and used to create a minimum spanning tree, developed utilizing BioNumerics version 7.6.3 (Applied Maths, Belgium). To explore the genetic relationships between CRISPR sequence variability and food type or serotype and CTs and food type, CTs and serotypes were displayed according to the results of the cluster analysis, respectively. Differences in CRISPR spacers comparing antibiotic-resistant and susceptible isolates were also examined. The spacer comparison and conversions to HEX color code were performed using CRISPRstudio software (Dion et al., 2018).

### Core Genome Phylogenetic Analyses

Among 257 *Cronobacter* isolates, whole genome sequences of 117 isolates were established based on core genome analyses and reported in our previous study (Zeng et al., 2017, 2018b). Next, a core genome ML phylogenetic tree was generated based on 287,220 nucleotides from concatenated 563 single-copy core genes sequences using FastTree (Price et al., 2009). The display and annotation of phylogenetic trees were performed using iTOL (Letunic and Bork, 2016).

## Results

### CRISPR Types

**FIGURE 2:**
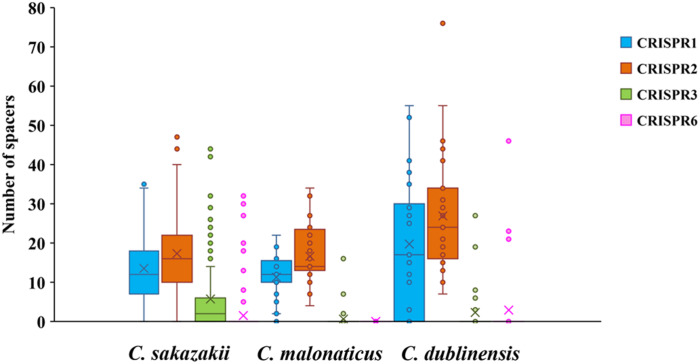
Number of spacers from four CRISPR arrays in *C. sakazakii*, *C. malonaticus*, and *C. dublinensis* strains.

Two hundred and fifty-seven isolates of *C. sakazakii*, *C. malonaticus*, and *C. dublinensis* were used to establish CRISPR subtyping method. The design of primers (sequences were listed in Table 1) and procedure of *Cronobacter* CRISPR typing method were shown in Figure 1. As shown in Figure 2, CRISPR1 and CRISPR2 loci were more conserved and active than others in all species; moreover, CRISPR2 had the largest average number of spacers. These results were in accordance with our previous study (Zeng et al., 2017, 2018b), and similar spacers were rarely detected among species, indicating intensive changes through adaptive acquisition and loss. In this study, the incidences of CRISPR1, CRISPR2, CRISPR3, and CRISPR6 in 161 *C. sakazakii* isolates were 99.4% (160/161), 99.4% (160/161), 57.1% (92/161), and 10.6% (17/161), respectively (Supplementary Data Sheet 1). Moreover, 1706 unique spacers were identified in *C. sakazakii* strains, and these were divided into 129 CTs; CT15 (*n* = 7) was the most prevalent followed by CT6 (*n* = 4). Regarding 65 *C. malonaticus* isolates (Supplementary Data Sheet 2), the incidences of CRISPR1, CRISPR2, CRISPR3, and CRISPR6 were 90.8% (59/65), 100% (65/65), 12.3% (8/65), and 0% (0/65), respectively. For this species, 487 unique spacers were identified in *C. malonaticus* strains, and they were divided into 42 CTs with CT23 (*n* = 8) being the most prevalent CT followed by CT2 (*n* = 4), CT3 (*n* = 4), and CT13 (*n* = 4). In *C. dublinensis* (Supplementary Data Sheet 3), the frequencies of CRISPR1, CRISPR2, CRISPR3, and CRISPR6 were 74.2% (23/31), 100% (31/31), 19.4% (6/31), and 4.9% (2/31), respectively. Further, 1361 unique spacers were identified in 31 *C. dublinensis* strains, and these belonged to 31 CTs. In these *C. sakazakii*, *C. malonaticus*, and *C. dublinensis* isolates, the discriminatory powers (a single numerical index of discrimination [D]) of CT were 0.9957, 0.9736, and 1.0000, respectively, indicating that there should be a 99.6, 97.4, and 100.0% probability that two unrelated isolates can be separated using the CT scheme. This method has comparable power to distinguish these species. The discriminatory powers of MLST for these species were 0.9669, 0.8986, and 0.9892, respectively, among these isolates. Thus, the CRISPR typing method showed better discriminatory power than MLST.

### Relationship Between CRISPR Sequence Variability and Food Type and Serotype and Antibiotic Resistance

**FIGURE 3:**
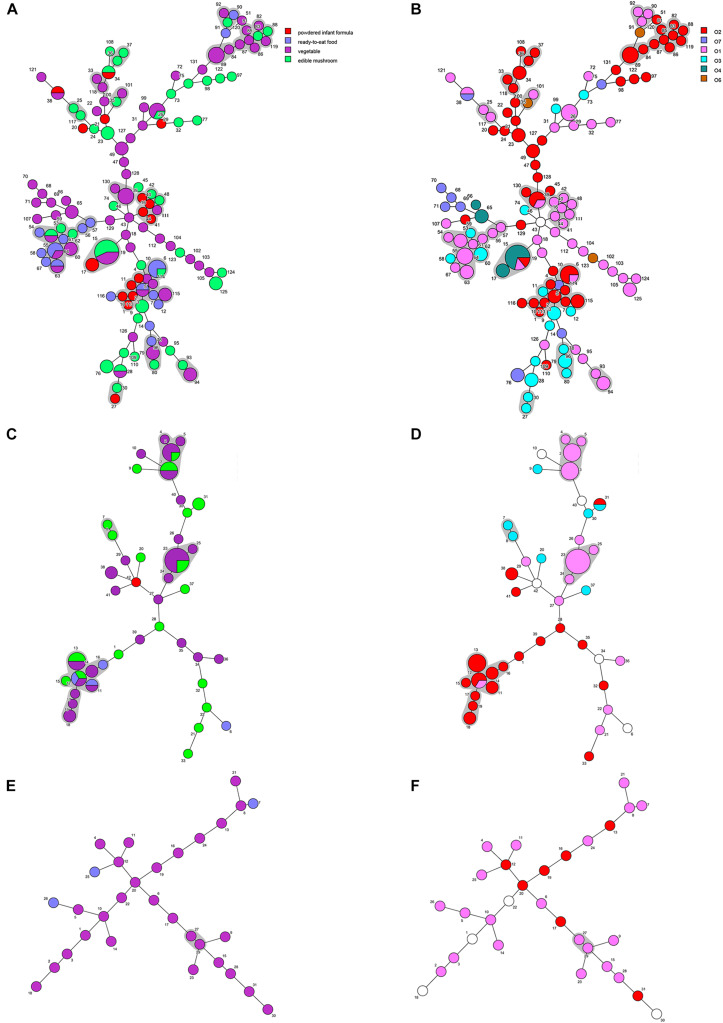
Minimum spanning tree of CRISPR data from 161 *C. sakazakii*, 65 *C. malonaticus*, and 31 *C. dublinensis* isolates. Minimum spanning tree of *C. sakazakii* **(A)**, *C. malonaticus* **(C)**, and *C. dublinensis* **(E)** isolates with color corresponding to each type of food indicated in the legend on the right side of **(A)**. Minimum spanning tree of *C. sakazakii* **(B)**, *C. malonaticus* **(D)**, and *C. dublinensis* **(F)** with color corresponding to each serotype indicated in the legend on the right side. Each circle represents one CRISPR type (CT), and the area of the circle corresponds to the number of isolates. The maximum distance between nodes in the same partition was set to 10.

Minimum spanning trees were generated using BioNumerics software to analyze the distribution of CTs among different types of food and their relationship with serotypes (Figure 3). *C. sakazakii*, *C. malonaticus*, and *C. dublinensis* strains isolated from vegetables showed higher CRISPR diversity than those from other types of food, and this was especially true for *C. dublinensis.* This was in accordance with previous studies showing a higher frequency and diversity of *Cronobacter* in vegetables compared to that in other types of food, supporting the contention that this species is plant-associated (Ueda, 2017; Ling et al., 2018; Silva et al., 2019). There was also a relationship between CT and serotype. As shown in Figure 2, when the maximum distance between nodes in the same partition was set to 10, CT6-, CT64-, CT15-, CT41-, and CT85-associated partitions were the five main partitions in *C. sakazakii* (Figure 3A). *C. sakazakii* serotype O2 was found among all strains of the CT85-associated partition and most strains of partition CT6; moreover, serotype O1 predominated the CT64- and CT48-associated partitions and most strains in the CT15-associated partition were serotype O4 (Figure 3B). For *C. malonaticus*, CT13-, CT23-, and CT2-associated partitions were the three major partitions (Figure 3C). All strains in CT23- and CT3-associated partitions were serotype O1, whereas serotype O2 was predominant in the CT13-associated partition (Figure 3D). Based on the limited number of isolates and high diversity of CRISPR sequences in *C. dublinensis*, there were no major partitions reported in this study, whereas O1 was the predominant serotype (Figure 3E). In accordance with previous studies, 96.9% (249/257) of isolates were resistant or intermediate to cephalothin, whereas most were susceptible to other antibiotics (Brandao et al., 2017; Ling et al., 2018). In total, there were three isolates resistant to two or more antibiotics in this study. Comparisons of CRISPR sequence variability between the resistant strains and other strains were also performed (Supplementary Data Sheets 1–3), there was no significant relationship between antibiotic resistance and CRISPR variability in *C. sakazakii*, *C. malonaticus*, and *C. dublinensis*.

### Accordance Among CRISPR Typing, MLST, and WGST

**FIGURE 4:**
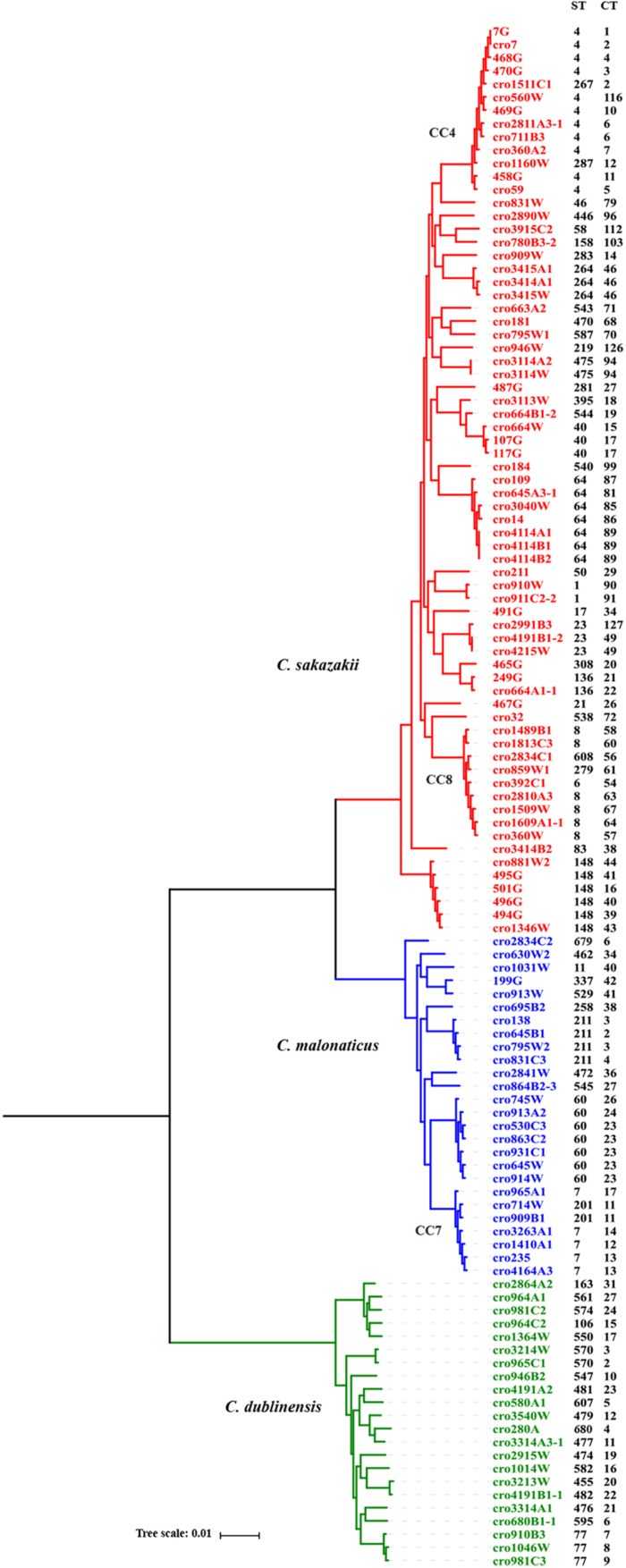
Phylogeny of 118 *C. sakazakii*, *C malonaticus*, and *C. dublinensis* strains inferred by whole genome sequences types (WGSTs). The STs and CRISPR types (CTs) of each isolate were listed on the right side and the CRISPR profiles of clonal complex 4 (CC4), CC8, CC7, ST148, ST60, and ST77 strains were also shown.

**FIGURE 5:**
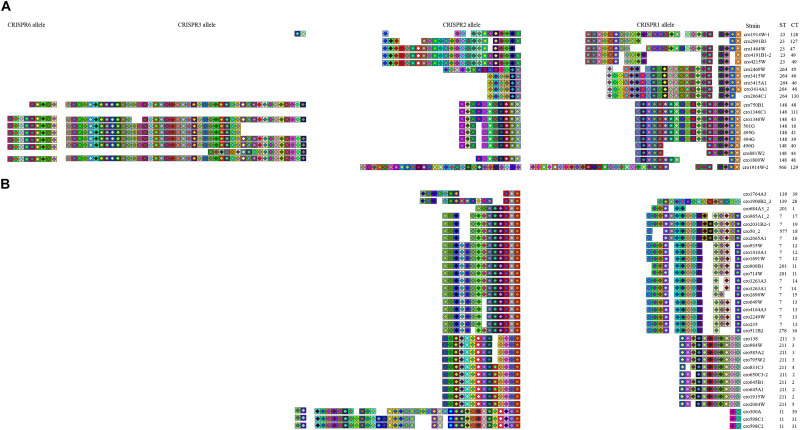
CRISPR spacer overview. Organization of spacer content of CRISPR alleles identified in 20 *C. sakazakii* isolates **(A)** and 33 *C. malonaticus* isolates **(B)**. Repeats were not shown in this figure, and only spacers were displayed. Color schemes were provided at the spacer level to visualize differences among isolates based on the software CRISPRStudio. Spacers are shown in the order of predicted acquisition in the locus (right, ancestral spacers; left, newly acquired spacers).

A core genome ML phylogenetic tree based on whole genome sequences of 117 strains was generated to evaluate the consistency between CRISPR typing and WGST. As shown in Figure 3, CRISPR profiles were conserved among phylogenetically related strains and these had a close relationship with ST types. At the same time, the strains with different STs but belonging to the same clonal complex (CC) also had similar CRISPR profiles and belonged to the same partition. *C. sakazakii* CC4, *C. sakazakii* CC8, and *C. malonaticus* CC7 were major pathogenic CCs in previous studies, all the strains in these CCs formed distinct clusters in the phylogenetic tree, and belonged to *C. sakazakii* CT6-, *C. sakazakii* CT64-, and *C. malonaticus* CT13-associated partitions, respectively. Moreover, this approach was found to distinguish the same ST into smaller units (Figures 4, 5). For example, seven ST64 isolates formed a small lineage in this phylogenetic tree, and three ST64 strains within the CT89-associated partition were more closely related than other strains of different CTs. At the same time, the phylogenetic distance between other ST64 strains was also in accordance with the differences in CRISPR spacer composition (Supplementary Data Sheet 1). The same phenomenon was observed for the ST23 strain (Figures 4, 5A). In *C. malonaticus* CC7, *C. malonaticus* ST7 isolates typed as CT12, CT14, CT13, and CT15 were more closely phylogenetically related to *C. malonaticus* ST211 isolates typed as CT11 than other ST7 isolates themselves (Figures 4, 5B), implying better accordance between the CRISPR typing method and WGST. However, there were also few inconsistent results, for example, *C. sakazakii* ST4 isolate cro7 and ST267 isolate cro1511C1 were *C. sakazakii* CT2, but cro7 have more closely phylogenetic relationship with another *C. sakazakii* ST4 isolate 7G. Combined with all the results, the CRISPR typing method shows better discriminatory power than MLST and has a better accordance with WGST.

### The Phylogenetic Information Inferred by Sequence Diversity in CRISPR Arrays

In addition to the good discriminatory power of CRISPR arrays in distinguishing *Cronobacter* strains, the phylogeny information conserved in the iterative spacer acquisition process can be used to infer common ancestry. The CRISPR alleles of *C. sakazakii* ST23, ST264, ST148, and ST566 strains are shown in Figure 5A. These ST isolates belonged to different CCs and no apparent close phylogenetic relationship among these STs was observed in Figure 3. In contrast to the high diversity of CRISPR spacers among these strains at four CRISPR loci, it was interesting to note that all of these strains harbored some conserved ancestral spacers in CRISPR1. There were seven ancestral spacers conserved in CRISPR1, and some ST23, ST264, and ST148 strains preserved all of these ancestral spacers. Moreover, one additional spacer inserted in the fourth and fifth ancestral spacers was detected in all ST148 strains. Unlike the first seven conserved spacer sequences, the newly incorporated spacers showed lineage specificity. The ancestral spacers might be important proof of lineage divergence. As shown in Figure 5B, *C. malonaticus* CC7, ST211, ST11, and ST139 isolates preserved one ancestral sequence in CRISPR2. ST139 strains had three to four ancestral spacers that were common with CC7 isolates, which suggests a closer phylogenetic relationship between these lineages.

## Discussion

The *Cronobacter* genus including seven species are opportunistic foodborne human pathogens that can cause rare but serious diseases in neonates and immune-compromised infants (Iversen et al., 2008; Kucerova et al., 2011; Joseph et al., 2012a). In our previous studies, *C. sakazakii*, *C. malonaticus*, and *C. dublinensis* were three prevalent species in food, however, we have never isolated *C. universalis* and *C. condimenti* strains (Xu et al., 2015; Ling et al., 2018; Li et al., 2019). In total, five *C. turicensis* and *one C. muytjensii* strains were isolated from vegetables and ready-to-eat foods (Xu et al., 2015; Ling et al., 2018) and we have successfully performed CRISPR arrays on these isolates using the same primers used for *C. sakazakii*. However, for the limited number of strains, whether these primers will be suitable for these species is unknown. Thus, we only constructed a CRISPR typing method for *C. sakazakii*, *C. malonaticus*, and *C. dublinensis* in this study.

In this study, CRISPR arrays were detected in all *Cronobacter* isolates; moreover, 1706, 487, and 1361 unique spacers were identified in 161 *C. sakazakii*, 65 *C. malonaticus*, and 31 *C. dublinensis* isolates. In accordance with a previous study (Zeng et al., 2018b), the number of CRISPR spacers in *C. dublinensis* isolates was greater than that in *C. sakazakii* and *C. malonaticus*. CRISPR1 and CRISPR2 were preserved in all three species and more active than other CRISPR loci; further, CRISPR3 was found in some strains of these species; however, CRISPR6 was only detected in some *C. sakazakii* and *C. dublinensis* strains (Supplementary Data Sheets 1–3). Whether there is a need to use four CRISPR loci for *C. malonaticus* CRISPR typing should be examined in the future using more isolates. Moreover, in these *C. sakazakii*, *C. malonaticus*, and *C. dublinensis* isolates, the discriminatory powers of the CRISPR typing method for all three species were comparable. According to our results, the CRISPR typing method shows better discriminatory power than MLST and has a better accordance with WGST. The largest outbreak of *C. sakazakii* occurred in a neonatal intensive care unit in France (1994), lasting over 3 months and claiming the lives of three neonates. A recent study used whole genome sequencing data of 26 isolates obtained from this outbreak to reveal relatedness (Masood et al., 2015). To examine the accuracy of CRISPR typing for the identification of pathogens in the *Cronobacter* outbreak, we downloaded these genome sequences and extracted CRISPR arrays for molecular typing. All *C. sakazakii* ST4, ST12, and ST13 strains belonged to CT2, CT50, and CT52, respectively. This was in accordance with the data obtained from the outbreak but had weaker discriminatory power compared to whole genome SNP analyses. In this study, 19 *C. sakazakii* ST4 strains isolated from several types of food in China were divided into 14 CTs including CT2, whereas four *C. sakazakii* ST13 isolates were divided into four CTs, but without CT52. Thus, the better discriminatory power of CRISPR typing could make it more useful than MLST to differentiate potential sources of *Cronobacter* outbreaks in the future.

Polarity exists as new spacers are always added to the proximal end of the CRISPR array; in addition, spacers at the leader distal end were found to be more ancient and were shared among phylogenetically related *Cronobacter* isolates. Spacer loss and gain make CRISPR elements the fastest evolving loci in *Cronobacter*, supporting previous speculation that CRISPR-Cas systems have an important impact on the evolution of this genus (Zeng et al., 2018b). CRISPR spacer variability in *Cronobacter* can divide an ST into smaller units and has better accordance with WGST than MLST. The CRISPR1 and CRISPR2 spacers in three species were more active, as shown in Figure 5, and some phylogenetically distant lineages were found to preserve some ancestral spacers at CRISPR1 or CRISPR2, respectively, although no similar spacers existed in other CRISPR loci. These ancestral spacers are important proof of lineage divergence; thus, CRISPR1 and CRISPR2 in *Cronobacter* can provide phylogenetic anchors reflecting common origins. Unfortunately, despite the extremely high variation in CRISPR spacer sequences of *Cronobacter*, many lineages had a unique CRISPR pattern, and no common ancestral spacers were found among these different clonal isolates. In summary, CRISPR diversity can be used to unfold a complete evolutionary story of strain divergence and relatedness, showing unique advantages compared to other genotyping methods.

The advantages of CRISPR-based genotyping methods have been demonstrated for some bacteria widely found in the food supply chain such as *Streptococcus thermophilus* (Horvath et al., 2008) and *Lactobacillus buchneri* (Briner and Barrangou, 2014). It can also be used for pathogenic strains like *Escherichia coli* (Yin et al., 2013; Barrangou and Dudley, 2016), *Salmonella* (Fabre et al., 2012; Li et al., 2014), *Clostridium difficile* (Andersen et al., 2016), and *Mycobacterium tuberculosis* (Streicher et al., 2007; Zhang et al., 2010). Finally, we also found a relationship between CT, ST, food types, and serotypes among *Cronobacter* isolates, and this phenomenon has also been found in other foodborne pathogens (Li et al., 2014, 2018; Bugarel et al., 2018).

## Conclusion

In conclusion, we developed a CRISPR typing method for *C. sakazakii*, *C. malonaticus*, and *C. dublinensis.* Compared to MLST, this new molecular method has greater power to distinguish similar strains and had better accordance with WGST. Compared to WGST, CRISPR typing is simpler and more affordable, and it could be useful for the identification of sources of *Cronobacter* outbreaks, in addition to performing microbial risk assessment during food processing. More importantly, CRISPR diversity can be used to infer the divergent evolution of *Cronobacter* and provide phylogenetic anchors reflecting common origins. In the future, it would be meaningful to generate a comprehensive *Cronobacter* complex database of CRISPR spacers for the global application of CRISPR typing, and pool the results of different research groups to explore the epidemiology and reservoirs of *Cronobacter* spp.

## Data Availability

The raw data supporting the conclusions of this manuscript will be made available by the authors, without undue reservation, to any qualified researcher.

## Author Contributions

HZ and QW conceived and designed the experiments. HZ, CL, WH, MC, TL, HW, and NL performed the experiments. HZ, JZ, SC, JW, and YD analyzed the data. HZ drafted the manuscript. QW supervised the project. All authors read and approved the final manuscript.

## Conflict of Interest Statement

The authors declare that the research was conducted in the absence of any commercial or financial relationships that could be construed as a potential conflict of interest.

## Footnotes

1 https://pubmlst.org/cronobacter/
