# Corrigendum: *Cronobacter sakazakii, Cronobacter malonaticus, and Cronobacter dublinensis* Genotyping Based on CRISPR Locus Diversity

**DOI:** 10.3389/fmicb.2020.01103

**Published:** 2020-06-04

**Authors:** Haiyan Zeng, Chengsi Li, Wenjing He, Jumei Zhang, Moutong Chen, Tao Lei, Haoming Wu, Na Ling, Shuzhen Cai, Juan Wang, Yu Ding, Qingping Wu

**Affiliations:** ^1^State Key Laboratory of Applied Microbiology Southern China, Guangdong Provincial Key Laboratory of Microbiology Culture Collection and Application, Guangdong Open Laboratory of Applied Microbiology, Guangdong Institute of Microbiology, Guangdong Academy of Sciences, Guangzhou, China; ^2^College of Food Science, South China Agricultural University, Guangzhou, China; ^3^Department of Food Science and Technology, Jinan University, Guangzhou, China

**Keywords:** *C. sakazakii*, *C. malonaticus*, *C. dublinensis*, CRISPR typing, multi-locus sequence typing, whole genome sequence typing

In the original article, there was an error. The annealing and extension time of PCR were wrong. The annealing time of PCR should be 5 s not 1 min, and extension time should be 4 min not 1 min, respectively.

A correction has been made to the section **Materials and Methods**, sub-section CRISPR PCR Amplification and Sequencing:

“58°C for CRISPR1 and 57°C for CRISPR2, CRISPR3, and CRISPR6 for 5 s, and 72°C for 4 min;”

The authors apologize for this error and state that this does not change the scientific conclusions of the article in any way. The original article has been updated.

